# Current Pharmacological Intervention and Medical Management for Diabetic Kidney Transplant Recipients

**DOI:** 10.3390/pharmaceutics13030413

**Published:** 2021-03-19

**Authors:** Theerawut Klangjareonchai, Natsuki Eguchi, Ekamol Tantisattamo, Antoney J. Ferrey, Uttam Reddy, Donald C. Dafoe, Hirohito Ichii

**Affiliations:** 1 Division of Transplantation, Department of Surgery, University of California, Irvine, Orange, CA 92868, USA; theerawutklang@gmail.com (T.K.); neguchi@hs.uci.edu (N.E.); ddafoe@hs.uci.edu (D.C.D.); 2 Department of Medicine, Faculty of Medicine, Ramathibodi Hospital, Mahidol University, Bangkok 10400, Thailand; 3 Division of Nephrology, Hypertension and Kidney Transplantation, Department of Medicine, University of California, Irvine, Orange, CA 92868, USA; etantisa@hs.uci.edu (E.T.); ferreya@hs.uci.edu (A.J.F.); ureddy@hs.uci.edu (U.R.)

**Keywords:** diabetes mellitus, kidney transplant, post-transplant diabetes mellitus (PTDM), new onset diabetes after transplantation (NODAT), cyclosporine, tacrolimus, dipeptidyl peptidase-4 (DDP-4) inhibitors

## Abstract

Hyperglycemia after kidney transplantation is common in both diabetic and non-diabetic patients. Both pretransplant and post-transplant diabetes mellitus are associated with increased kidney allograft failure and mortality. Glucose management may be challenging for kidney transplant recipients. The pathophysiology and pattern of hyperglycemia in patients following kidney transplantation is different from those with type 2 diabetes mellitus. In patients with pre-existing and post-transplant diabetes mellitus, there is limited data on the management of hyperglycemia after kidney transplantation. The following article discusses the nomenclature and diagnosis of pre- and post-transplant diabetes mellitus, the impact of transplant-related hyperglycemia on patient and kidney allograft outcomes, risk factors and potential pathogenic mechanisms of hyperglycemia after kidney transplantation, glucose management before and after transplantation, and modalities for prevention of post-transplant diabetes mellitus.

## 1. Introduction

Kidney transplantation (KT) is currently the most promising form of renal replacement therapy [1]. KT improves patient survival, quality of life [2], and cost-effectiveness [3] when compared to dialysis. Based on Organ Procurement and Transplantation Network (OPTN) data, as of 23 February 2021, KT has become the standard practice, with almost half a million people having undergone this procedure in the United States between 1988 and 2021. The utilization of numerous immunosuppressive agents has improved graft and patient survival, leading to long-term recipient care [4]. Immunosuppressive agents including corticosteroids, cyclosporine, and tacrolimus influence glucose metabolism and increase the risk of diabetes mellitus (DM).

Data analysis from the United States Renal Data System (USRDS) reports pretransplant diabetes occurs in 24% of patients and total occurrence of post-transplant diabetes mellitus (PTDM) in 16% and 24% of cases at 1 and 3 years after KT, respectively [5]. Pretransplant DM has be associated with graft failure and death following KT, with cardiovascular events being the cause of death in over 60% of these patients. However, death-censored graft loss and acute graft rejection are comparable between patients with and without pretransplant DM [6]. On the other hand, PTDM has been associated with increased rate of graft rejection, graft failure, and death, contrasted with recipients without DM [7]. This review will first discuss the terminology and diagnosis of pretransplant and post-transplant DM. Second, the effect of transplant-related hyperglycemia on cardiovascular disease, mortality, and graft-related complications will be explained. Third, changes in glucose metabolism and factors that contribute to hyperglycemia after KT will be discussed. In particular, the effect of diabetogenic immunosuppressive agents and chronic viral infection on post-transplant hyperglycemia will be discussed. Fourth, we will discuss glucose management in pretransplant and post-transplant periods and review antidiabetic agents that have been evaluated for their efficacy and safety in KT recipients. Finally, we will discuss strategies for prevention of PTDM.

## 2. Impact of Diabetes Mellitus on Kidney Transplant

### 2.1. Terminology and Diagnosis

Patients with pre-existing or pretransplant DM are defined as those with a clinical diagnosis of DM prior to KT. Pretransplant DM increases the risk of KT graft failure and mortality [6]. Patients who present with hyperglycemia post-transplant are classified as either new onset diabetes after transplantation (NODAT) or PTDM. NODAT is a term for patients who develop new onset of DM following KT and excludes patients with undiagnosed pre-existing diabetes, as well as post-transplant temporary hyperglycemia that is usually caused by high-dose steroid induction. NODAT is seen as a complication of solid organs’ transplantation. A cross-sectional study demonstrates that 8% of KT candidates have undiagnosed DM and 37% of those have prediabetic condition [8]. As a result of the prevalence of undiagnosed pretransplant DM, NODAT is replaced by PTDM. PTDM is characterized as newly diagnosed DM, present but undetected before KT. The subclassification of diabetes post-transplantation into NODAT and PTDM is important because of the prevalence of undiagnosed pretransplant DM. This may be due to several reasons. First, pretransplant DM is often undiagnosed due to the influence of chronic kidney disease on the metabolism and clearance of insulin. Second, oral glucose tolerance testing, which is the preferred and more accurate method for diagnosing DM, is not routinely evaluated in KT candidates. Third, since the waiting time for deceased donor KT has been getting longer, some KT candidates may develop undiagnosed DM during this period.

Diagnosis guidelines for PTDM are fasting plasma glucose ≥ 126 mg/dL on more than one occasion, random plasma glucose ≥ 200 mg/dL with symptoms, or 2 h plasma glucose of ≥200 mg/dL following a 75 g oral glucose tolerance test (OGTT), which is the preferred evaluation method. Hemoglobin A1C (HbA1C) ≥ 6.5% can only be used to screen PTDM at 45 days following KT due to potential confounding factors in the early post-transplant period [9]. Early post-transplant anemia, dynamic function of kidney allograft, and use of iron and erythropoietin stimulating agents have been shown to have an effect in HbA1C regardless of glycemic change [9]. Additionally, stress hyperglycemia is very common (occurring in about 90% of recipients) during the early post-KT phase [10]. Therefore, the diagnosis of PTDM should only be made once the patient has been stabilized on maintenance immunosuppressive agents without infection or rejection. Of note, PTDM diagnosis has no “end date”. If a KT recipient is diagnosed with DM 1, 5, or 10 years later, it is still entitled to be named PTDM.

Since PTDM and NODAT do not take into consideration the population of patients in the prediabetic stage, new terminology has been created to completely encompass the entire at-risk population: pretransplant diabetes, NODAT, and PTDM. Diagnosis of transplant-associated hyperglycemia (TAH) includes PTDM, impaired fasting glucose (IFG), and impaired glucose tolerance (IGT) for the general population, according to the American Diabetes Association (ADA) and the World Health Organization (WHO) (Table 1).

### 2.2. Impact of Transplant-Associated Hyperglycemia on Cardiovascular Disease and Mortality

Both pretransplant DM and PTDM have been shown to adversely affect patient survival [11]. Several studies have linked PTDM to increased risk of cardiovascular (CV) disease [12,13,14]. Registry data collected by USRDS demonstrated that after diagnosis of PTDM, the risk of post-transplant myocardial infarction increased by 60% [14]. The incidence of CV events in patients with normoglycemia was significantly greater among patients with IFG and PTDM. Additionally, rising plasma glucose above 100 mg/dL was correlated with rising CV risk at any time after the first month following KT [12]. Supporting these findings, data from the Organ Procurement and Transplant Network/United Network for Organ Sharing (OPTN/UNOS) revealed that pretransplant DM is associated with increased risk of all-cause and CV deaths. Part of the high death rate in recipients with PTDM can be attributed to infectious complications [5]. On the other hand, NODAT had a statistically insignificant trend toward increased risk of all-cause and CV mortality during the first year [15]. 

### 2.3. Association of Transplant-Associated Hyperglycemia and Kidney Allograft-Related Complications

In addition to increased mortality, PTDM is also associated with graft loss and death-censored graft loss [5]. There are several potential mechanisms in which PTDM contributes to graft loss. First, PTDM may causes diabetic nephropathy in the kidney allograft. Second, DM with metabolic syndrome may lead to impaired vascular health and high blood pressure. On the other hand, acute graft rejection or dysfunction may result in hyperglycemia because patients may require higher doses of diabetogenic immunosuppressive agents (e.g., corticosteroid, tacrolimus, and cyclosporine) [16]. 

### 2.4. Glucose Metabolism after Kidney Transplantation

Hyperglycemia is common in both DM and non-DM patients in the early post-transplant phase. The distinct diurnal pattern of hyperglycemia is predictable, with a marked tendency towards higher glucose levels in the afternoon (2–3 p.m.) and in the evening (7–8 p.m.). Patients with pre-existing DM have higher glycemic variability in the first 5 days following KT than patients without DM. This may reflect the effect of the induction therapy with high-dose glucocorticoids. The glycemic variability and control improved after KT over 3–6 months [17]. The kidney plays an important role in the metabolism and clearance of insulin. Insulin is filtered by the glomeruli and reabsorbed in the proximal tubule. The insulin clearance rate is reduced in individuals with glomerular filtration rate (GFR) of less than 40 mL/min per 1.73 m^2^ and the insulin half-life is increased when GFR falls below 20 mL/min per 1.73 m^2^.

Interestingly, patients with PTDM exhibited both worse glycemic control and variability than non-transplanted patients with type 2 DM (T2DM) [18]. Pathophysiology of PTDM varies from that of T2DM. In contrast with T2DM in non-transplanted subjects, NODAT is primarily thought to be a result of impaired insulin secretion from the pancreatic beta-cell rather than a reduction in peripheral insulin sensitivity [19]. Moreover, impaired glucose uptake in muscle and adipose tissue, increased production of glucose, inadequate incretin signals between the intestine and pancreas, and decreased suppression of glucagon are also observed in PTDM [20,21]. Although PTDM does not report neurotransmitter dysfunction in the brain that regulates appetite, the use of corticosteroids in recipients of KT can stimulate appetite and food intake that will result in weight gain over the long term [22]. Increased renal gluconeogenesis and glucose reabsorption at proximal tubules occur in T2DM but are not yet established in PTDM [16,23]. 

### 2.5. Factors Affecting Glucose Metabolism in Kidney Allograft Recipients

USRDS data analysis identified several modifiable risk factors for PTDM, such as obesity, hepatitis C infection, and type of initial maintenance immunosuppressive agents used. In the recipients treated with tacrolimus, the risk of PTDM is 53% greater, while the use of azathioprine and mycophenolate mofetil is associated with 16% and 22% reduced risks for PTDM, respectively. Other risk factors for developing PTDM include older age, African American race, Hispanic ethnic background, male donor, human leukocyte antigen (HLA) mismatches, and less than a college education [5]. In addition, family background of DM, male recipient gender, deceased donor kidney, acute rejection history, certain HLA (HLA A30, B27, and B42), hypomagnesemia, post-transplantation proteinuria, cytomegalovirus infection, and metabolic syndrome components (obesity, pretransplant IFG/IGT, hyperlipidemia, and hypertension) have been associated with higher risk for developing PTDM [24,25,26]. Suggested risk factors which affect the metabolism of glucose following KT can be categorized as non-modifiable and potentially modifiable, as shown in Table 2.

## 3. Potential Mechanisms of Diabetogenic Immunosuppressive Agents and Chronic Viral Infection

### 3.1. Corticosteroids

Several mechanisms have been implicated in corticosteroid-induced hyperglycemia. First, corticosteroids may contribute to hyperglycemia through decreasing peripheral sensitivity to insulin, increasing liver gluconeogenesis, increasing lipolysis, and reducing muscle and adipose tissue glucose uptake. Second, it may inhibit the secretion and production of insulin from pancreatic beta-cells and induce apoptosis of beta-cells. Third, corticosteroids may enhance the effect of glucagon secreted by pancreatic alpha-cells, which increases the endogenous synthesis of glucose. Fourth, it may reduce the effect of incretin by decreasing glucagon-like peptide-1 (GLP-1) hormone secreted from the intestine, thereby inducing more long-term appetite and accompanying weight gain.

### 3.2. Calcineurin Inhibitors (CNIs) (Cyclosporine and Tacrolimus)

CNIs are well-known to induce hyperglycemia in a dose-dependent manner [27]. This may be due to reduced insulin secretion since CNIs have been shown to reduce expression of the insulin gene and close Adenosine triphosphate (ATP)-sensitive potassium channels. Tacrolimus tends to cause more frequent and severe damage to the islet cells when compared to cyclosporine. CNI toxicity to islet cells is considered to be reversible. Furthermore, in patients with hepatitis C virus infection, PTDM occurred more often in tacrolimus-treated patients when compared with cyclosporine A. This was not the case in non-infected individuals [28].

### 3.3. Mammalian Target of Rapamycin (mTOR) Inhibitors (Sirolimus and Everolimus)

Sirolimus has been shown to cause dose-dependent hyperglycemia and to reduce insulin sensitivity in the short term. Furthermore, sirolimus decreases pancreatic beta-cell insulin secretion and beta-cell proliferation [29]. Everolimus is a newer mTOR inhibitor, therefore there are few studies evaluating its diabetogenic effects. However, it is suggested that everolimus, similarly to sirolimus, reduces insulin sensitivity [22]. 

### 3.4. Other Immunosuppressive Agents

The antimetabolites, azathioprine and mycophenolate mofetil (MMF), have not been demonstrated to affect glucose metabolism. Moreover, the USRDS data analysis illustrates that azathioprine and MMF are associated with a lower risk for developing PTDM [14]. Belatacept is a humanized fusion protein that inhibits the costimulatory pathway by blocking T cell activation. Belatacept-based regimens do not appear to increase NODAT following KT compared to cyclosporine-based regimens [30]. 

### 3.5. Hepatitis C Virus Infection

Chronic hepatitis C virus infection mostly influences peripheral insulin sensitivity through reducing hepatic glucose absorption and glycogenesis [31]. One study indicated that hepatitis C virus infection was independently associated with an increase in insulin resistance by 62%, while no difference was seen in beta-cell function between the presence and absence of hepatitis C virus infection [32]. 

### 3.6. Cytomegalovirus (CMV) Infection

**Figure 1:**
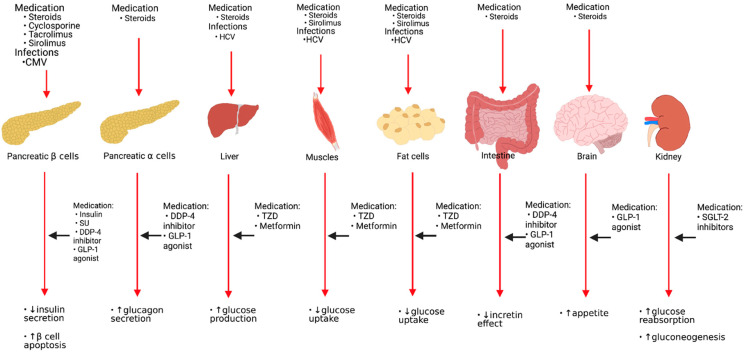
Pathogenesis and risk factors of glucose metabolism following kidney transplantation and sites of actions of antidiabetic agents. HCV: Hepatitis C virus, CMV: Cytomegalovirus virus, SU: Sulfonylureas, DDP-4: Dipeptidyl Peptidase-4, GLP-1: Glucagon-like peptide-1, TZD: Thiazolidinedione, SGLT-2: Sodium-glucose cotransporter type 2.

Asymptomatic and symptomatic CMV infection is a risk factor for developing PTDM. CMV infection raises the incidence of NODAT in the first three months following KT by 4-fold [33]. One proposed mechanism of CMV-induced DM is the release of proinflammatory cytokines that may induce apoptosis or cause functional disturbances of pancreatic beta-cells [27]. Pathogenesis and risk factors of glucose metabolism following KT and sites of actions of antidiabetic agents are shown in Figure 1.

## 4. Glucose Management in KT

**Figure 2:**
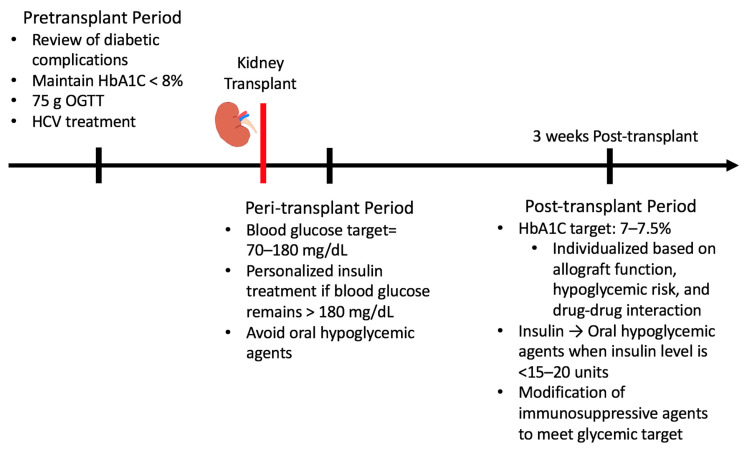
Glucose management in pre-, peri-, and late post-transplant periods.

Proper glucose management in KT recipients is critical to minimize adverse events following transplantation. Different guidelines should be followed during the pretransplant, peri-transplant, and post-transplant periods (Figure 2.)

### 4.1. Pretransplant Period

Pretransplant assessment for patients with pre-existing DM should include a review of the history of diabetic complications, including microvascular (diabetic retinopathy and diabetic neuropathy) and macrovascular complications (cardio- and cerebro-vascular comorbidities), documentation of glucose levels, history of hyperglycemic and hypoglycemic events, and use of anti-diabetic drugs. It is suggested that pretransplant HbA1c is maintained at less than 8% for pre-existing DM candidates with hemodialysis treatment, as poor glycemic control pretransplant increases all-cause mortality, including cardiovascular events. Moreover, poor glycemic control is linked to higher rates of infection, chronic inflammation, and macrovascular complications, thus shortening patient survival. However, counterintuitively, pretransplant HbA1c level is not a predictor of complications related to KT [34].

In addition to review of past medical history, physical examination and laboratory testing should be used to screen for risk factors for metabolic syndrome and cardiovascular disease: 75 g OGTT should be undertaken for DM screening before patients are placed on the KT waiting list. At some transplant centers, however, 75 g OGTT may be impractical due to the cost. Patients with abnormal OGTT prior to transplantation should be instructed on lifestyle changes, including diet modification, regular physical activity, and weight loss. Furthermore, pretransplant HCV infection treatment should be considered to minimize the risk of post-transplant DM. Lastly, immunosuppressive regimens should be selected to take into consideration against the risk of developing hyperglycemia as well as the risk of rejection of the graft.

### 4.2. Peri Post-Transplant Period

The pancreatic beta-cells are exposed to several hyperglycemic stressors during the immediate post-transplant period, which are caused by the KT operation itself involving pain, blood loss, and rapid hemodynamic changes, and high-dose corticosteroids administration and the addition of immunosuppressive agents such as cyclosporine or tacrolimus. There were no specific glycemic targets for this period. Tight glycemic control (blood glucose target, 70–110 mg/dL) in the first 72 h after KT was associated with increased hypoglycemic events and it may increase the risk of episodes of rejection compared to conventional glycemic control (blood glucose target, 70–180 mg/dL) [35]. Therefore, it seems appropriate for KT recipients to follow current guidelines for the control of in-patient glucose.

Insulin treatment should be initiated if glucose level remains above 180 mg/dL for persistent hyperglycemia. For patients in both intensive and non-intensive care units, a target blood glucose level of 140–180 mg/dL is recommended once insulin is started [36]. Insulin regimen following transplantation should be individualized based on overall health of the patient and nutritional intake. Hospitalized patients who are critically ill should use continuous intravenous insulin infusion and frequent blood glucose check from every 30 min to every 2 h. For patients in non-intensive care units, insulin regimen should be started based on nutritional intake following operation. Non-critically ill hospitalized patients with poor oral intake should be treated with basal insulin or a basal plus bolus insulin correction regimen, while those with good oral intake should be treated with basal, prandial, and correction components [36]. Blood glucose level should be checked before each meal in patients with good oral intake or every 4–6 h in patients who are not eating. Once patients have a stable oral intake of food, they may transition to subcutaneous insulin therapy. When converting to subcutaneous insulin therapy, total daily subcutaneous insulin dose should be approximately 60–80% of the daily dose of insulin infusion. Subcutaneous basal insulin should be administered 2–4 h before the intravenous insulin is stopped. The general strategy for hospitalized DM also avoids the use of oral hypoglycemic agents due to fear of side effects and lack of effectiveness in this setting [36]. 

### 4.3. Late Post-Transplant Period

Late post-transplant is described as more than 3 weeks following KT. Glucose management in the late post-transplant period is important to reduce the risk of mortality associated with PTDM. A study that conducted OGTT in KT recipients 10 weeks post-transplant found that each 1 mmol/L (18 mg/dL) increase in 2 h plasma glucose was associated with a 5% increased risk of death from any cause and 6% increased risk of death from cardiovascular events. Additionally, this study demonstrated that 2 h plasma glucose is superior to fasting post-kidney transplantation plasma glucose in predicting all-cause and cardiovascular mortality in recipients of kidney transplantation [37]. 

Though PTDM pathogenesis is different from T2DM, PTDM treatment will follow the standard glycemic management for T2DM in this time period. According to the ADA, the target HbA1c goal for the general DM population is less than 7%, the less stringent target is less than 8%, and the more stringent target is less than 6.5% for certain individuals depending on the duration of DM, life expectancy, comorbidities, hypoglycemic events, patient preference, and medical support network [38]. For recipients of KT, the American Society of Transplantation and the American Society of Transplant Surgeons advise target HbA1c to be around 7–7.5%. Additionally, HbA1c of less than 6% should be avoided, particularly if hypoglycemic events are common in the patient [39]. A multicenter retrospective cohort study in Korea supports these recommendations for glucose control following KT, even though the exact range of HbA1c does not concord. This study showed that both strict control of glucose as well as poor control of glucose were associated with kidney allograft failure, which was defined as composite of graft dysfunction that required new renal replacement therapy following transplantation or patient death. The HbA1c range associated with the best graft outcome was 7.6–8.6% [40]. Target HbA1c for the post-KT period should be individualized based on kidney allograft function, hypoglycemia risks, and drug–drug interactions.

Insulin tapering and bridging to oral hypoglycemic agents can be considered when the insulin level is less than 15–20 units per day after the first 1–3 months following KT. Lifestyle modification should be recommended to control DM, including healthy diabetic diet, regular exercise, weight reduction, and management of sleep and stress. Additionally, modification of immunosuppression should be considered if glycemic control fails to achieve therapeutic targets. Rapid steroid taper, steroid-sparing protocols, tacrolimus conversion to cyclosporine therapy, and avoidance of combination therapy with CNIs and mTOR inhibitors should be considered in these cases. Before manipulating their immunosuppressive therapy, clinicians must consider patients’ immune history, such as panel reactive antibody status, race, and prior transplant [25]. 

## 5. Antidiabetic Agents

Although many classes of drugs have been used for T2DM, only a few antidiabetic agents have been validated for treating hyperglycemia in KT recipients. The choice of antidiabetic agents should take into consideration the general health status of the patient, functions of the kidney allograft, associated medical disorders, adverse drug events, drug–drug interactions, and expenses. This individualized approach refers to the ABCDE of diabetes treatment in KT recipients:

A = Allograft function and adverse drug events

B = Body weight

C = Comorbidities

D = Drug–drug interactions

E = Expenses

The potential advantages and disadvantages of antidiabetic agents for KT recipients are shown in Table 3.

### 5.1. Insulin

Nearly all KT patients may experience hyperglycemia due to corticosteroid dosing or other immunosuppressive agents. Hyperglycemia exacerbates ischemia or reperfusion injury, inflammation, and oxidative stress. Glycemic regulation after KT is challenging. Insulin therapy is preferred over oral hypoglycemic agents in hospitalized patients. A randomized controlled trial in 3-day post-KT patients with DM or impaired glucose tolerance demonstrated that conventional insulin regimen (subcutaneous isophane or glargine and a part insulin using and blood glucose target 70–180 mg/dL) lowered the incidence of kidney allograft rejection episodes and severe hypoglycemic events when compared to an intensive insulin regimen (intravenous regular insulin using and blood glucose target 70–110 mg/dL). However, the two treatment groups did not show any statistical difference in the prevalence of delayed graft function and severe hyperglycemia [35]. Therefore, under the current general practice recommendations for inpatient blood glucose management, blood glucose level will range between 140 and 180 mg/dL in hospitalized patients who use insulin therapy [36]. 

### 5.2. Metformin

The advantages of metformin are weight-neutral, low risk of hypoglycemia, low cost, cardio-protection, and few drug–drug interactions. Metformin does not undergo significant hepatic metabolism and therefore is mostly excreted unchanged in the urine. Additionally, metformin is a substrate for multiple membrane transporters in the liver, kidney, and intestine, and thus, is rarely involved in drug–drug interactions [41]. However, metformin can cause lactic acidosis, especially in the early post-transplant period. Data from the Scientific Registry of Transplant Recipients shows that nearly 5% of patients with pre-existing DM received metformin in the first year after KT. The use of metformin is not related to any negative outcomes of the patient or the graft. In fact, metformin use is associated with about a 60% lower all-cause, malignancy-related, and infection-related mortality when compared to insulin use without metformin. In addition, metformin alone has no-significant trends toward decreased risk of acute rejection, graft failure, and cardiovascular death when compared with insulin use without metformin. Although the recommendation is that the use of metformin should be avoided if GFR is less than 30 mL/min per 1.73 m^2^, a study revealed that 1.5% of diabetic KT recipients with GFR < 30 mL/min per 1.73 m^2^ are exposed to metformin in the first year. This study concluded that the use of metformin in selected KT recipients may be safe [42]. A small randomized controlled trial showed that there is no serious adverse drug event and lactic acidosis episode among KT recipients, that have estimated GFR > 30 mL/min per 1.73 m^2^. Abdominal pain, indigestion, and metallic taste are reported from recipients after the use of metformin, which resolves after discontinuation [43]. 

### 5.3. Sulfonylureas and Glinides

Sulfonylureas and glinides stimulate insulin secretion from pancreatic beta-cells by closing ATP-sensitive potassium channels in the plasma membrane of the beta-cell. Sulfonylureas are metabolized by cytochrome P450 2C9 (CYP2C9) and CYP2C19, and the resultant metabolites are further broken down by the kidneys. Therefore, in the case of renal impairment and increased risk of prolonged hypoglycemia, sulfonylureas may accumulate. Trimethoprim, metronidazole, and fluconazole are inhibitors of CYP2C9, which can cause increased sulfonylurea levels and increased risk of hypoglycemia.

Repaglinide is metabolized extensively by CYP enzymes, including CYP3A4 and CYP2C8, with minimal renal elimination. The CYP3A4 also metabolizes cyclosporine, tacrolimus, and sirolimus [41]. Cyclosporine raises repaglinide level in healthy volunteers by inhibiting CYP3A4 [44]. One study reported that repaglinide may be an effective treatment option for KT recipients with PTDM as it successfully lowered blood glucose, similarly to rosiglitazone treatment. Mild hypoglycemia was reported in 23% of patients and liver enzymes level did not change significantly during repaglinide use in chronic viral hepatitis. Moreover, there was no significant change in cyclosporine, tacrolimus, and sirolimus levels following repaglinide use in this study [45]. Some concerns of sulfonylureas and glinides treatment are weight gain, hypoglycemia, cardiovascular safety, and beta-cell exhaustion. Sulfonylureas may cause progressive beta-cell failure after the initial 18 months of treatment and therefore, they do not prevent or delay the loss of pancreatic beta-cells in DM [46]. 

### 5.4. Thiazolidinediones or Glitazones

Thiazolidinediones act on the peroxisome proliferator-activated receptor (PPAR)-gamma, enhancing insulin sensitivity in peripheral tissues including muscle, fat, and liver. Due to cardiovascular safety concerns, rosiglitazone was removed from the market. Thus, currently, pioglitazone is the only thiazolidinedione on the market [47]. Pioglitazone is a substrate of CYP2C8, and to a lesser degree to CYP3A4, but has no effect on CYP enzyme [48]. Therefore, pioglitazone should not result in drug–drug interactions with immunosuppressive agents in KT recipients. In a randomized controlled trial, addition of pioglitazone to insulin in diabetic KT recipients not only reduced HbA1c and daily insulin requirements, but also reduced cardiovascular inflammatory markers, including erythrocytes sedimentation rate, C-reactive protein, and high-sensitivity C-reactive protein. In addition, pioglitazone administration did not change the level of interleukin-18, the proinflammatory cytokine associated with kidney rejection and metabolic syndrome when compared to the placebo group. Furthermore, although 10% of the patients in the pioglitazone groups reported mild to moderate lower-extremity edema, there was no significant difference in body weight between the two groups [49]. Some reported adverse effects of pioglitazone are weight gain, particularly when combined with sulfonylureas or insulin, fluid retention, which results in leg edema and heart failure, increased incidence of bone fractures, and bladder cancer.

### 5.5. Dipeptidyl Peptidase-4 (DDP-4) Inhibitors or Gliptins

The DDP-4 inhibitors include sitagliptin, vildagliptin, linagliptin, saxagliptin, alogliptin, and gemigliptin. Gliptins inhibit the degradation of incretin hormones, including GLP-1 and glucose-dependent insulinotropic polypeptide, by inhibiting DDP-4 enzymes. This results in increased insulin synthesis and secretion, suppression of glucagon secretion, inhibition of gastric emptying, and suppression of appetite and dietary intake [50]. DDP-4 inhibitors have also shown positive effects on the cardiovascular system, and have been recently associated with blood pressure control [51]. Moreover, DDP-4 inhibitors have theoretically been shown to protect the pancreatic beta-cells by activating GLP-1 [52]. Some favorable characteristics of gliptins are weight-neutral, low risk of hypoglycemia, and low risk of drug–drug interactions.

A randomized controlled trial reported that vildagliptin safely and efficiently improved 2 h postprandial glucose and HbA1c at 3 months following treatment, compared to baseline and placebo groups. There was no difference in insulin sensitivity between the two groups in fasting and postprandial states, suggesting that the therapeutic effect of vildagliptin was most likely a result of improved beta-cell function. No serious adverse drug events, kidney, and liver function changes have been reported, and there was no difference in tacrolimus and cyclosporine levels between vildagliptin and placebo groups [53]. 

In a randomized controlled cross-over study in KT recipients with PTDM, sitagliptin therapy increased first- and second-phase insulin secretion and insulin sensitivity compared to control. In addition, fasting plasma glucose and postprandial glucose were reduced following treatment with sitagliptin. Sitagliptin treatment did not result in significant differences in body weight, blood pressure, C-reactive protein, liver function, and trough concentrations of cyclosporine, tacrolimus, everolimus, and mycophenolate relative to control [54]. In another study, however, when added on to metformin, sitagliptin treatment resulted in average weight loss of 0.4 kg, while insulin glargine treatment resulted in average weight gain of 0.8 kg in KT recipients with PTDM. This effect of sitagliptin on weight loss was statistically significant. Additionally, the reduction of HbA1c and fasting plasma glucose, and the occurrence of hypoglycemia, were comparable between sitagliptin and insulin glargine when added-on to metformin. Therefore, this study suggests that considering that metformin treatment alone cannot achieve an HbA1c of less than 7% in KT recipients with PTDM with stable allograft function for more than 6 months after KT, sitagliptin may be a preferential second-line therapy to prevent weight gain during PTDM treatment [55]. Supporting these findings, a study in a small cohort of KT recipients with PTDM has shown that sitagliptin is effective as both a single agent or in combination with other antidiabetic agents. Additionally, sitagliptin was also well-tolerated and did not alter renal function and immunosuppressive levels for 12 months following initiation of sitagliptin [56]. 

A retrospective study in real-world settings reported that linagliptin is effective for glycemic control in DM patients following KT. Only minimal side effects and no significant change in tacrolimus level, kidney function, or body weight were observed after 24 weeks of linagliptin therapy [57]. In the immediate post-KT period, the combination of linagliptin plus basal bolus insulin regimen provided better glycemic control with lower insulin demands and less serious hypoglycemia than the basal bolus insulin regimen alone [58]. Therefore, linagliptin may help to decrease glucose variation as a risk factor for hypoglycemia in hospitalized KT patients treated with basal bolus insulin regimen. In a retrospective study comparing the efficacy of DDP-4 inhibitors (sitagliptin, vildagliptin, and linagliptin) in KT recipients with T2DM, the linagliptin group significantly decreased HbA1c compared to vildagliptin and sitagliptin groups [59]. 

Lastly, in terms of metabolism of DDP-4 inhibitors, saxagliptin and gemigliptin are metabolized by CYP3A4 and therefore can be used with immunosuppressive agents. All gliptins are excreted predominantly by the kidney as an unchanged parent compound, excluding linagliptin and gemigliptin, which are excreted mainly by biliary excretion and therefore, do not need renal dose adjustment [60,61]. 

### 5.6. Sodium-Glucose Cotransporter Type 2 (SGLT2) Inhibitors or Gliflozins

Canagliflozin, dapagliflozin, and empagliflozin are SGLT2 inhibitors. Gliflozins increase the excretion of urinary glucose by reducing the reabsorption of glucose in the proximal tubules. SGLT2 inhibitors are an insulin-independent mechanism, producing a therapeutic effect by increasing the excretion of urinary glucose without causing hypoglycemia. SGLT2 inhibitor has some benefits in reducing body weight and blood pressure. However, SGLT2 inhibitors are associated with adverse events, such as genitourinary tract infection, lower limb amputation, bone fractures, euglycemic diabetic ketoacidosis, acute kidney injury from diuretics, contrast media, nonsteroidal anti-inflammatory drugs (NSAIDs), and volume depletion [62]. They should therefore be avoided in individuals with a history of recurrent urinary tract infections and those who have previously demonstrated a propensity to volume depletion. Drug elimination of SGLT2 inhibitors occurs primarily by hepatic metabolism via glucuronidation to inactive metabolites and to a lesser degree, by renal elimination as a parent drug. CYP enzymes play a relatively limited role in the metabolism of gliflozin.

Gliflozins are a substrate of efflux pump P-glycloprotein (P-gp) [41]. In healthy volunteers, cyclosporine inhibits P-gp, and therefore increases the level of canagliflozin. However, due to the high safety margin of SGLT2 inhibitors, this is unlikely to cause hypoglycemia [63]. A pilot study that examined the efficacy and safety of canagliflozin in recipients with T2DM or PTDM showed that 6 months of add-on canagliflozin therapy resulted in lowering of HbA1c, body weight, and systolic blood pressure compared to baseline. Kidney function and tacrolimus level were not significantly affected, hypoglycemia was not found in any cases, and urinary tract and genital infections were not increased [64]. Similar to canagliflozin, a randomized controlled trial reported that empagliflozin safely improved glucose control in KT recipients with PTDM compared to placebo. The study showed a significant decrease in HbA1c and body weight in the 24-week empagliflozin treatment group compared to placebo, with no significant differences in adverse events, kidney functions, and levels of tacrolimus, cyclosporine, and everolimus between the groups. However, there was also no significant difference in insulin secretion and sensitivity between the two groups [65]. 

## 6. Prevention of PTDM

### 6.1. Lifestyle Modification

Despite receiving lifestyle modification leaflets, glucose metabolism deteriorates in KT recipients. Intensive modification of lifestyle including dietician referral, exercise programs, and weight loss advice can reverse glucose intolerance when compared to a passive approach [66]. A prospective cohort study demonstrated that a Mediterranean style diet may help to reduce the risk of NODAT and all-cause mortality in KT recipients. Mediterranean style diet is high in consumption of whole grains, legumes, fruit, vegetables, olive oil, and fish, and limited in intake of dairy products and meats. The high antioxidants, fiber, magnesium, and unsaturated fatty acids in the Mediterranean style diet are considered to improve insulin sensitivity and pancreatic beta-cell function, and decrease inflammation and endothelial dysfunctions [67]. Thus, a healthy diet is important as a secondary prevention for a patients undergoing KT. The current nutrition guideline for KT recipients is 45–60% of total energy intake allocated to carbohydrates, 10–30% to protein, and 20–30% to fats, with less than 10% from saturated fats. Three key micronutrients, phosphorus (1250 mg/day), magnesium (360 mg/day for females and 410 mg/day for males), and vitamin D (600 IU/day), help improve insulin sensitivity and have renoprotective effects [68]. 

### 6.2. Basal Insulin Injection to Oral Therapy

The TIP study (Treat-to-target trail of basal insulin to oral therapy in post-transplant hyperglycemia) revealed that KT recipients treated with basal insulin (isophane insulin for evening blood glucose > 140 mg/dL) during the first 3 weeks after KT exhibited significantly lower HbA1C level compared to those treated with standard-of-care control (antihyperglycemic agents for blood glucose > 180–250 mg/dL) at 3 months and after 3 months over a 1 year follow-up. In addition, the basal insulin treatment group had enhanced pancreatic beta-cell function and reduced risk of developing DM after 1-year post-transplantation by 73% [69]. 

### 6.3. Pharmacological Intervention in KT Recipients

One pilot randomized controlled trial showed that metformin showed no efficacy for prevention of PTDM in KT recipients. Nonetheless, this study revealed that metformin use in KT recipients with eGFR greater than 30 mL/min per 1.73 m^2^ is safe and has good tolerability without serious adverse events [43]. 

A randomized controlled trial demonstrated that both vildagliptin and pioglitazone treatment significantly reduced 2 h plasma glucose level at 3 months after initiation of treatment and decreased HbA1C compared to a placebo group in KT recipients with impaired glucose tolerance. Therefore, in addition to lifestyle modification, pharmacological intervention with vildagliptin or pioglitazone in KT recipients with newly diagnosed PTDM may prove beneficial [70]. 

## 7. New Therapeutic Approaches

As discussed earlier, pathophysiology of NODAT and PTDM differs from that of T2DM in that post-transplant hyperglycemia is considered to be a result of impaired insulin secretion by pancreatic beta-cells instead of reduced insulin sensitivity. Therefore, new therapeutic approaches focusing on preservation of beta-cells may prove beneficial in reducing the risk of developing NODAT and PTDM. It has long been known that some anti-diabetic medications, especially those in the thiazolidinediones class, protect beta-cells through reducing beta-cell dedifferentiation and apoptosis through PPAR-gamma activation [71,72]. More recently however, the use of nuclear factor erythroid 2-related factor 2 (Nrf2) activators for beta-cell preservation show promising results. The Nrf2 pathway plays a significant role in protecting beta-cells against various stressors, including endogenous and exogenous oxidants. A study investigating the effect of Nrf2 activation by dh404 on human pancreatic islets found upregulation of common antioxidant enzymes including NAD(P)H: Quinone oxidoreductase, Heme oxygenase 1 (HO-1), glucose 6 phosphate dehydrogenase (G6Pd), sulfiredoxin-1, and thioredoxin reductase1 (TXNRD1) [73]. Considering that hyperglycemia has been linked closely with redox imbalance in beta-cells, Nrf2 activators may provide beta-cell protection during the inevitable hyperglycemia evident in the immediate post-transplant period. In vitro, several Nrf2 pathway activators, including dimethyl fumarate (DMF), oltipraz, dh404, curcumin, and sulforaphane, have been shown to preserve beta-cell function and mass under different stressors in human and/or rodent beta-cells [74,75,76,77]. In a clinical trial, 9 months of curcumin treatment successfully reduced the number of prediabetic patients who progressed to T2DM [77]. In vitro, studies using human pancreatic beta-cells showed that curcumin protected beta-cells through upregulation of common antioxidants and increased insulin secretion [78,79]. Furthermore, although it has only been studied in vitro, alongside cyclosporine, curcumin synergistically inhibited peripheral blood lymphocytes of KT recipients experiencing rejection [80]. Supporting these findings, another in vitro study demonstrated that curcumin and resveratrol, another Nrf2 activator, suppressed action of T cells and B cells through inhibition of proliferation, antibody production, and lymphokine secretion [81]. However, curcumin has been shown to significantly inhibit the activity of CYP3A4, which may alter metabolism of immunosuppressive medication [82]. In fact, there has been a reported case on supratherapeutic tacrolimus level in a liver transplant recipient who consumed a high dose of curcumin through ingestion of turmeric with food [83]. However, the patient in this case reported to have taken 15 spoonsful of turmeric/day, significantly above the recommended ½ to 1 teaspoon/day (2.5–5 g/day). Therefore, further studies need to be conducted to determine whether lower-dose curcumin could be used alongside immunosuppressive medications or whether curcumin could be used to decrease the dose of tacrolimus prescribed to transplant recipients. All in all, use of Nrf2 activators for beta-cell preservation to reduce the risk of developing NODAT and PTDM holds tremendous preventative and therapeutic potential.

## 8. Conclusions

KT is an effective treatment for end-stage renal disease. After KT, glucose metabolism is affected by various factors. Pretransplant and post-transplant DM have been associated with increased graft failure and mortality. All candidates for KT should be informed that newly diagnosed DM rates are high during the first year and patients with pre-existing DM will have glycemic control worsened after KT. DM screening before KT may require 75 g OGTT. Target HbA1c should be less than 8% for candidates with pre-existing DM and about 7–7.5% for post-KT. Modification of lifestyle should be recommended, including dietician referral, exercise programs, and weight reduction, to control DM. In addition, treatment for HCV infection and selection of immunosuppressive regimens should be considered to minimize the risk of post-transplant DM. Although there are many anti-hyperglycemic drugs currently available, there is currently little reported clinical data to guide the physician regarding the advantages and risks of antidiabetic drugs in KT recipients. Large-scale randomized controlled trials of these drugs in DM management following KT will prove crucial to determine optimal treatment guidelines in these patients.

## Figures and Tables

**Table 1:** Criteria for the diagnosis of diabetes and prediabetes ^a.^

Test	Normal	Prediabetes		Diabetes
		IFG ^b^	IGT ^c^	
FPG ^d^	<100 mg/dL	100–125 mg/dL	-	≥126 mg/dL
	(5.6 mol/L)	(5.6–6.9 mol/L)		(7.0 mmol/L)
2 h plasma glucose ^e^	<140 mg/dL	-	140–199 mg/dL	≥200 mg/dL
	(7.8 mmol/L)		(7.8–11.0 mol/L)	(11.1 mmol/L)
Random plasma glucose plus symptoms ^f^	-	-	-	≥200 mg/dL
				(11.1 mmol/L)
HbA1C ^g^	<5.7%	5.7–6.4%		≥6.5%
	(39 mmol/mol)	(39–47 mmol/mol)		(48 mmol/mol)

^a^ A confirmatory laboratory test based on measurements of venous plasma glucose must be done on another day in the absence of unequivocal hyperglycemia accompanied by acute metabolic decompensation. ^b^ IFG is impaired fasting glucose, ^c^ IGT is impaired glucose tolerance. ^d^ Fasting plasma glucose (FPG) is defined as venous plasma glucose after no caloric intake for at least 8 h. ^e^ 2-h plasma glucose is venous plasma glucose 2 h after ingestion of 75 g anhydrous glucose dissolved in water. ^f^ Random plasma glucose is any time of day without regard to time since last meal. The classic symptoms of diabetes include polyuria, polydipsia, and unexplained weight loss. ^g^ HbA1C should be performed in a laboratory using a method that is National Glycohemoglobin Standardization Program certified and standardized to the Diabetes Control and Complications Trial assay.

**Table 2:** Suggested risk factors affect glucose metabolism after kidney transplantation.

Non-Modifiable Risk Factors	Potentially Modifiable Risk Factors
Age ≥ 45 years Male recipient African American, Hispanic Family history of diabetes mellitus Human leukocyte antigen (HLA) mismatch HLA A30, B27, B42 Acute rejection history Male donor Deceased donor	**Metabolic Syndrome Components**
	Obesity (Body mass index > 30 kg/m^2^)
	Pretransplant IFG/IGT
	Hyperlipidemia
	Hypertension
	**Viral Infection**
	Hepatitis C virus
	Cytomegalovirus
	**Immunosuppressive Agents**
	Corticosteroids
	Tacrolimus
	Cyclosporine
	Sirolimus
	**Others**
	Hypomagnesemia
	Proteinuria

**Table 3:** The potential advantages and disadvantages of antidiabetic agents.

Antidiabetic Agents	Advantages	Disadvantages/Comments
Insulin	-Theoretically no ceiling effect -No renal dose adjustment -Less drug–drug interaction	-Risk of hypoglycemia -Weight gain -Expense depends on type of insulin -Need education about administration
Metformin	-Low risk of hypoglycemia -Weight-neutral -Cardio-protection	-Avoid in glomerular filtration rate (GFR) < 30 mL/min per 1.73 m^2^ due to risk of lactic acidosis. -Gastrointestinal intolerance and vitamin B12 deficiency.
Sulfonylureas and Glinides	-Low cost	-Renal dose adjustment depends on types of drugs (no dose adjustment in gliplizide and repaglinide). -Risk of hypoglycemia, especially when used with trimethoprim, metronidazole, and fluconazole. -Weight gain -Beta-cell exhaustion
Pioglitazone	-No renal dose adjustment -Low risk of hypoglycemia -Less drug–drug interaction -Low cost	-Weight gain, especially when used with insulin or sulfonylureas. -Adverse effects including heart failure, leg edema, bone fracture, and bladder cancer.
Dipeptidyl Peptidase-4 Inhibitors	-Low risk of hypoglycemia -Weight-neutral -Less drug–drug interaction, except saxagliptin -Safe in cardiovascular disease, except saxagliptin -Possible beta-cell preservation	-May require renal dose adjustment depending on types of drugs. -High expense
Sodium-glucose cotransporter type 2 inhibitors	No hypoglycemia Reduced body weight, blood pressure, and cardiovascular events. Less drug–drug interaction	-Avoid in GFR less than 30 mL/min per 1.73 m^2^ for canagliflozin and empagliflozin. -Avoid in GFR less than 45 mL/min per 1.73 m^2^ for dapagliflozin. -Adverse effects including genitourinary tracts infection, dehydration, euglycemic diabetic ketoaciodosis, and bone fracture. -High Expense

## Data Availability

Data Sharing Not Applicable.

## Abbreviations

KT	Kidney Transplantation
OPTN	Organ Procurement and Transplantation Network
DM	Diabetes Mellitus
USRDS	United States Renal Data System
PTDM	Post-transplant diabetes mellitus
NODAT	New onset diabetes after transplantation
OGTT	Oral glucose tolerance test
TAH	Transplant-associated hyperglycemia
IFG	Impaired fasting glucose
IGT	Impaired glucose tolerance
ADA	American Diabetes Association
WHO	World Health Organization
FPG	Fasting plasma glucose
HbA1C	Hemoglobin A1C
CV	Cardiovascular
GFR	Glomerular Filtration Rate
T2DM	Type 2 DM
HLA	Human leukocyte antigen
GLP-1	Glucagon-like peptide 1
CNI	Calcineurin inhibitors
mTOR	Mammalian target of rapamycin
CMV	Cytomegalovirus
PPAR	Proliferator-activated receptor
DDP-4	Dipeptidyl Peptidase 4
SGLT2	Sodium-glucose cotransporter type 2
NSAIDs	Nonsteroidal anti-inflammatory drugs
P-gp	P-glycloprotein
Nrf2	Nuclear factor erythroid 2-related factor
HO-1	Heme oxygenase 1
G6PD	Glucose 6 phosphate dehydrogenase
TXNRD1	Thioredoxin reductase 1
DMF	Dimethyl Fumarate
